# Infliximab Induces Increase in Triglyceride Levels in Psoriatic Arthritis Patients

**DOI:** 10.1155/2011/352686

**Published:** 2011-07-24

**Authors:** Karla R. Castro, Nádia E. Aikawa, Carla Gonçalves Saad, Júlio C. B. Moraes, Ana C. Medeiros, Licia Maria H. Mota, Clovis A. A. Silva, Eloísa Bonfá, Jozélio F. Carvalho

**Affiliations:** ^1^Rheumatology Division, Hospital das Clínicas da Faculdade de Medicina da Universidade de São Paulo (CEDMAC), 01246-903 São Paulo, SP, Brazil; ^2^Pediatric Rheumatology Unit, Hospital das Clínicas da Faculdade de Medicina da Universidade de São Paulo (CEDMAC), 01246-903 São Paulo, SP, Brazil; ^3^Rheumatology Division, University Hospital of Brasília, Universidade de Brasília, 71660020 Brasília, DF, Brazil; ^4^Disciplina de Reumatologia da FMUSP, Avenidu Dr. Arnaldo, 455, 3 Andar, sala 3190, 01246-903 São Paulo, SP, Brazil

## Abstract

*Objectives*. To evaluate lipid profile changes after anti-TNF therapy in patients with psoriatic arthritis (PsA). *Methods*. Fifteen PsA patients (eight polyarticular, four oligoarticular, two axial, and one mutilating) under infliximab were included. None had dyslipoproteinemia or previous statin use. Total cholesterol (TC) and its fractions, inflammatory markers, and prednisone use were evaluated. *Results*. The comparisons of lipid levels between baseline and after three months (3M) of anti-TNF therapy showed that there was a significant increase in mean triglycerides (117.8 ± 49.7 versus 140.1 ± 64.1 mg/dL, *P* = 0.028) and VLDL-c (23.6 ± 10.5 versus 28.4 ± 13.7 mg/dL, *P* = 0.019) levels. In contrast, there were no differences in the mean TC (*P* = 0.28), LDL-c (*P* = 0.42), and HDL-c (*P* = 0.26) levels. Analysis of the frequencies of each lipid alteration at baseline and at 3M were alike (*P* > 0.05). Positive correlations were found between VLDL-c and CRP (*r* = 0.647, *P* = 0.009) and between triglycerides and CRP (*r* = 0.604, *P* = 0.017) levels at 3M. ESR reduction was observed after 3M (*P* = 0.04). Mean prednisone dose remained stable at beginning and at 3M (*P* = 0.37). *Conclusion*. This study demonstrated that anti-TNF may increase TG and VLDL-c levels in PsA patients after three months.

## 1. Introduction

Psoriatic arthritis (PsA) is a serious chronic inflammatory arthropathy of unknown cause that is associated with cutaneous psoriatic lesions [1]. It is a chronic illness that occurs in 6–39% of patients with psoriasis [2–5], depending of the number of participants. It is calculated that 5 to 7% of the patients with psoriasis will develop peripheral arthritis, and the clinical forms distinguished are oligoarticular, polyarticular, axial, and mutilating [1].

The treatment of PsA is conducted through the use of nonhormonal anti-inflammatory (NSAID) agents that are utilized in routine treatment and provide evident improvements in joint pain [6]. Like corticosteroids, prednisone in low doses can eventually be used in cases of persistent peripheral arthritis [7]. In cases where patients are nonresponsive to NSAID, the drug of first choice is methotrexate, in weekly doses (orally or intramuscularly), until control of the disease is obtained [8]. A new era in the treatment of rheumatic diseases began with the development of TNF-*α*-inhibitors, where TNF-*α* is a cytokine that performs an important role in inflammatory diseases, which have shown satisfactory results for both joint and skin diseases [9, 10]. Adverse effects, including infections and lipid profile changes, have been related to this treatment for rheumatoid arthritis and ankylosing spondylitis [11, 12]. However, there are only case reports and one prospective study that evaluated the effects of these new medications on the lipid profiles of individuals with PsA, with very few patients and different periods of followup [13, 14].

Due to the paucity of studies, as well as the contradictory results in previous findings, the aim of this study was to evaluate lipid profile changes pre- and post-anti-TNF therapy treatment of PsA patients.

## 2. Material and Methods

### 2.1. Patients

This prospective study included 15 patients presenting with PsA (Moll and Wright classification [15]): eight patients with polyarticular, four with oligoarticular, two with axial, and one with mutilating forms of the disease, who were treated with anti-TNF therapy (etanercept and infliximab) and assessed during follow-up appointments at the High Cost Medication Dispensation Center (CEDMAC) of the Hospital das Clínicas da Faculdade de Medicina da Universidade de São Paulo. Clinical and demographic data were collected through review of the electronic charts and medical consultations before the beginning of anti-TNF treatment and after three months of treatment. 

The exclusion criteria included: previous diagnoses of dyslipidemia, diabetes mellitus, renal failure, liver disease, and use of medications that could interfere with lipid metabolism (statins, fibrates, and thiazide). A venous blood sample was collected at fast for laboratory analysis before and after three months of anti-TNF treatment.

This study was approved by the Research Ethic Committee of our University Hospital and an informed consent form was obtained from all participants.

### 2.2. Lipid Profile

Total cholesterol (TC) and triglycerides (TGs) in the serum samples were measured enzymatically (Boehinger-Mannhim, Argentina and Merck, Germany, resp.) with an RA 1000 Analyzer (Technicon Instruments Corp) [16, 17]. The high-density lipoprotein cholesterol (HDL-c) levels were obtained after precipitation of the very-low-density lipoprotein cholesterol (VLDL-c) from the serum and from the low-density lipoprotein cholesterol (LDL-c) using phosphotungstic acid and magnesium chlorate [18]. VLDL-c and LDL-c levels were estimated since all the samples had triglyceride levels lower than 400 mg/dL [17]. The VDL-c levels were determined using the ratio of the triglyceride levels/five (TG/5), and the LDL-c levels were estimated using the following equation [18]: TC = HDL-c + TG/5 + LDL-c.

### 2.3. Inflammatory Activity Tests

The C-reactive protein dosage (CRP) (Roche Diagnosis, Indianapolis, USA) was assessed by nephelometry. The erythrocyte sedimentation rate (ESR) was obtained using the modified Westergreen technique.

### 2.4. Statistical Analysis

The results were presented in either means and standard deviations or percentages. Statistical analysis was performed using the GraphPad InStat version 2.00 program and Microsoft Excel, and Student's t-test to and the Fisher's exact test were used to compare the continuous and categorical results, respectively. In all the statistical tests, the level of significance was set at 5% (*P* < 0.05).

## 3. Results

From a total of 26 PsA patients, 11 were excluded due to previous history of dyslipidemia or drugs use that interfere with lipid levels and one due to etanercept use. The mean age of the 15 patients included in this study was 41.9 ± 9.3 years, and 53% were male and 93% were Caucasians. The average duration of the disease was 14.6 ± 7.9 years. The patients included in this study were using either infliximab (*n* = 15) or etanercept (*n* = 1). 

Twelve (80%) patients experienced improvement in skin and/or arthritis at 3 months. Analysis of inflammatory markers revealed that there was a significant reduction in ESR values between preadministration of anti-TNF and after three months (25.4 ± 22.4 versus 12.8 ± 18.5 mm/1st hour, *P* = 0.038). A trend to lower CRP was observed in these two moments (17.6 ± 18.4 versus 10.8 ± 15.9 mm/1st hour, *P* = 0.058). Regarding prednisone use, the dose was low and stable throughout the study, with a variation in the mean of equivalent doses between the beginning of the study and after three months (*P* = 0.24) (Table 1). 

The total cholesterol (*P* = 0.30), LDL-c (*P* = 0.39) and HDL-c (*P* = 0.26) levels did not showed significant differences in comparison to the values at the baseline and at three months after treatment, respectively. In relation to the TG levels (126.1 ± 50.1 versus 146.5 ± 61.4 mg/dL, *P* = 0.024) and VLDL-c levels (25.3 ± 10.6 versus 29.7 ± 12.5 mg/dL, *P* = 0.016), there was a significant increase in the levels of these lipids. The frequencies of dyslipidemia at baseline and after three months were alike (*P* > 0.05) (Table 2). TC/HDL-c ratio did not differ in the two studied periods. Reinforcing, positive correlations were found between VLDL-c and CRP levels (*r* = 0.647, *P* = 0.009), as well as between TG and CRP levels (*r* = 0.604, *P* = 0.017) at 3 months.

Glucose levels did not alter significantly in the period studied (88.2 ± 18.3 versus 90.6 ± 20.9 mg/dL, *P* = 0.32).

## 4. Discussion

This study demonstrated a significant increase in the triglyceride and VLDL levels after three months of anti-TNF therapy used in patients with PsA. Advantages of the present study were to exclude patients with previous diagnoses of dyslipidemia, diabetes mellitus, and use of statins and to include only patients with established diagnosis of PsA. 

As observed in our study, the TG and VLDL-c levels increased significantly. The TC, HDL-c, LDL-c, and CRP levels did not have significant variations during anti-TNF treatment. A significant reduction in ESR levels was observed when comparing the pre- and postperiods of anti-TNF treatment, indicating a good response to TNF blockers. In another study, Cauza et al. observed a significant increase in the TG levels and a decrease in HDL-c concentrations in eight patients with PsA after infliximab therapy [13]. Conversely, Sparnakis et al. found a significant increase in HDL-c levels during the evaluation and only a significant increase in TC in the first month of evaluation. There was a significant ESR and CRP reduction only in the third and first month after infliximab infusions in ten patients with PsA [14]. The remarkable association of VLDL-c and CRP levels as well as between TG and CRP demonstrated herein reinforces the role of inflammation and disease activity in Takayasu patients. Indeed, CRP is a pentraxin synthesized mainly in the liver in response to mediators of inflammation, and there is substantial evidence that CRP besides has a strong predictive power for cardiovascular events [19]. 

Similar studies with rheumatoid arthritis (RA) patients also presented contradictory results. In a study by Popa et al., they sought to evaluate whether anti-TNF treatment modified the cardiovascular risk in 33 patients with RA. They assessed the lipid profiles of patients before and at two weeks after the first adalimumab dose. They stated that the treatment reduced inflammation and improved the cardiovascular profile of these patients by improving their lipid profiles (HDL-c increase and LDL-c decrease) [11]. Another study evaluated 34 patients with RA, using the three TNF blockers, and found an increasing of TC, HDL-c, and LDL-c levels, with no changes in TG levels or the atherogenic index after approximately six months of anti-TNF treatment [20]. Soubrier et al. investigated the lipid profiles of 29 RA patients before and after anti-TNF treatment for 14 weeks and did not show any lipid profile change in these patients [21].

In a more recent study, Popa et al. investigated the effects of anti-TNF treatment on the lipid profiles of 55 RA patients after one year of treatment. They could demonstrate that the treatment modified the plasma concentrations in the lipoproteins of these patients. There was an increase in HDL-c concentrations and no changes in the atherogenic index during the first two weeks of treatment. Nevertheless, the beneficial effects were not sustained over time; there were eventually TC and LDL-c increases even though the HDL-c levels did not change for twelve months. This finding suggested a worsening of the lipid profiles and an increase in the atherogenic index of these patients, which could increase their cardiovascular risk [22].

TNF-*α* promotes expression of adhesion molecules in endothelial cells, which initiates an inflammatory cascade in the vessel wall [23, 24]. Additionally, TNF-*α* directly interferes with the metabolic routes of TGs and cholesterol [25, 26]. TNF-*α* administration results in an elevation of TG levels and a reduction of HDL-c levels [27].

Hypertriglyceridemia is a well-known factor of cardiovascular risk: high TG levels predict the subsequent occurrence of coronary artery disease [28]. The implementation of strategies for control of hypertriglyceridemia, as well as LDL-c levels, reduces coronary artery disease risk [29]. In fact, patients with PsA present an increased prevalence of atherosclerotic disease, sometimes subclinical [30]. Cardiovascular disease and its risk factors, including dyslipidemia, are more common among patients with PsA than in control groups [31]. 

It is clinically evident that PsA patients without cardiovascular risk factors or cardiovascular disease have an elevated prevalence for macrovascular disease, which is represented by an increased carotid intima-media thickness [32]. This thickness independently correlates with activity parameters in PsA and traditional risk factors for atherosclerosis [33]. These data corroborate the idea that special attention and strict control of the risk factors for atherosclerotic diseases in patients with PsA are necessary [30].

In summary, this study was able to demonstrate that patients with PsA who received anti-TNF therapy showed increased TG and VLDL levels after a three-month period. Larger prospective long-term studies are necessary in order to assess the impact of these lipid alterations in the cardiovascular disease in that group of patients.

##  Conflict of Interests

The author declares that there is no conflict of interests.

## Figures and Tables

**Table 1:** Inflammatory markers, prednisone use/dose, and levels at baseline and at three months after starting anti-TNF therapy.

Variable	Baseline	Three months	*P* value
ESR (mean ± SD, mm in the first hour)	25.4 ± 22.4	12.8 ± 18.5	0.038
CRP (mean ± SD, mg/L)	17.6 ± 18.4	10.8 ± 15.9	0.058
Prednisone use, *n* (%)	7 (44)	7 (44)	1.0
Prednisone dose (mean ± SD, mg/day)	2.0 ± 2.5	2.8 ± 5.2	0.24

TNF: tumor necrosis factor, ESR: erythrocyte sedimentation rate, and CRP: C-reactive protein.

**Table 2:** Lipid profile at baseline and at three months after starting anti-TNF therapy in psoriatic arthritis patients.

Variable	Baseline	Three months	*P* value
Total cholesterol (mg/dL)	157.1 ± 32.9	163.9 ± 33.8	0.30
HDL-c (mg/dL)	49.7 ± 15.4	45.1 ± 11.0	0.26
LDL-c (mg/dL)	91.7 ± 24.4	92.7 ± 18.9	0.39
VLDL-c (mg/dL)	25.3 ± 10.6	29.7 ± 12.5	**0.016**
Triglycerides (mg/dL)	126.1 ± 50.1	146.5 ± 61.4	**0.024**
Total cholesterol >200 mg/dL	1 (6)	0	1.0
HDL-c <40 mg/dL(male)/ <50 mg/dL(female)	5 (37)	5 (31)	1.0
LDL-c > 130 mg/dL	0	0	1.0
VLDL-c > 40 mg/dL	1 (6)	2 (12)	1.0
Triglycerides > 150 mg/dL	5 (31)	8 (50)	0.47

TNF: tumor necrosis factor, HDL-: high-density cholesterol, LDL: low-density cholesterol, and VLDL-c: very-low-density cholesterol.
